# Construction Notes

**Published:** 1897-03-20

**Authors:** 


### CONSTRUCTION NOTES.

### New District Fever Hospital at Falkirk.

The hospital buildings which have been erected as the new district fever hospital by the Eastern District Committee of the Stirling County Council near Camelon. They consist of four large blocks, viz., an administrative block, a scarlet fever block, an enteric fever block, and laundry buildings. Occupying the centre position, the administrative block faces the public road, the scarlet fever and enteric fever blocks lying to the east and west of the same, with the laundry behind. The administrative block is a two-storey building, and contains medical officer's room, house accommodation for the matron and nurses, with a room for the staff and four bedrooms in the upper storey for the accommodation of the nurses. Attached to the building there are also two observation wards for the admission and supervision of doubtful cases, with lavatory accommodation in each. Ample provision is also made for the kitchen, scullery, and pantry requirements of the building, and, situated below in the basement, is the heating chamber for the whole building. The scarlet fever block contains one large and one smaller ward, the former containing eight beds, and the latter four beds. Between the two wards is placed the nurses' room, with specially constructed windows, so that the nurse can overlook the wards. The nurses' room is also provided with kitchen range and other requirements for the cooking purposes of the wards. Attached to each ward there is bath-room and all lavatory accommodation, as well as a portable bath for moving about the wards when required. There is also in connection with the building a convalescent ward with one bed. The enteric fever ward consists also of two wards, and is exactly similar to the scarlet fever block, with the exception that each of the wards contains four beds, and there is no convalescent room attached. In both blocks the buildings have been so constructed, and the beds so arranged, that in cases of emergency provision can be made for a greater number of patients than they are meantime intended for. The laundry buildings include rooms for the reception and distribution of infected articles of clothing, in connection with which a patent disinfector has been fitted up by Mr. A. B. Reck, of Copenhagen. There is also attached a mortuary, van-shed, boiler-house, and ambulance wagon shed. The total cost, including furnishings of this very complete hospital, is estimated at about £7,500. The architects are Messrs. A. and W. Black.

